# The brief introduction to organizational citizenship behaviors and counterproductive work behaviors: a literature review

**DOI:** 10.3389/fpsyg.2023.1181930

**Published:** 2023-09-13

**Authors:** Qianqian Fan, Walton Wider, Choon Kit Chan

**Affiliations:** ^1^Faculty of Business and Communications, INTI International University, Nilai, Negeri Sembilan, Malaysia; ^2^International Education College, Hebei Finance University, Baoding, China; ^3^Department of Mechanical Engineering, Faculty of Engineering and Quantity Surveying, INTI International University, Nilai, Negeri Sembilan, Malaysia

**Keywords:** organizational performance, organizational citizenship behavior, counterproductive work behavior, economic productivity, influencing factors

## Abstract

This paper presents a literature review on the topic of organizational performance. The study conceptualizes the overall performance of the organization as comprising of organizational citizenship behaviors (OCB) and counterproductive work behaviors (CWB). While there are numerous research studies on OCB, not many have focused on how OCB and CWB affect organizational performance simultaneously. The paper provides an explanation of the OCB and CWB concepts, followed by the primary research and focus of the study. The article presents a comprehensive framework for understanding the meanings of OCB and CWB, along with an internal hierarchy. This framework will serve as a beneficial resource for working managers, academics, and researchers, who seek to optimize economic productivity through improved understanding and management of OCB and CWB.

## Introduction

Employees play a direct or indirect role in numerous factors that affect the operational results of an organization, by “shaping the organizational, social, and psychological context that serves as the catalyst for task activities and processes.” This behavior is referred to by some scholars as Organizational Citizenship Behavior (OCB) or Counterproductive Work Behavior (CWB), both of which have been the subject of numerous psychological and management studies (Shah et al., 2022). According to these scholars, OCB is associated with an ethical organizational working environment and corporate sustainability performance (Fein et al., 2023). In contrast, CWB represents intentionally destructive conduct aimed at harming an organization’s legitimate interests (Lee, 2020). In previous research, many scholars have explained employee behaviors using Blau’s (1964) social exchange theory and the theory of Person-Organization Fit (POF) (Kristof-Brown et al., 2005). The former elucidates the interaction among attitudes, perceptions, and behaviors, interpreting employee behaviors as a two-way communication between the individual and the organization (Yıldız et al., 2015). The latter serves as a predictor of certain positive behaviors (e.g., OCB) and negative behaviors (e.g., CWB). In studying constructive workplace behaviors, researchers have distinguished between OCB and CCB (Compulsory Citizenship Behaviors). They have also identified the differential effects of various antecedents, including equity sensitivity, Chinese tradition, and job stress (Yildiz et al., 2023). In research on destructive deviant workplace behaviors, these behaviors have been labeled with various terms that share similar meanings, such as counterproductive workplace behaviors (CWB) (Yıldız et al., 2015). Furthermore, Yıldız and Alpkan (2015) proposed a comprehensive model to analyze these destructive deviant workplace behaviors. They also introduced individual and organizational antecedents of negative behaviors, including POF, careerism, participative decision-making, and alienation. Current findings suggest that the more positive an employee’s perceptions are of OCB, the less likely they are to engage in negative behavior. Most recent research in this field supports these findings (Hossein and Somayeh, 2018; Jiang et al., 2022; Fein et al., 2023). These behaviors are shaped by the intent and direction of targeted actions (Neuhoff, 2020).

## The definition of OCB and CWB

The concept of OCB was formally recognized by Organ (1988), who introduced it as a variable that could enhance organizational effectiveness (Yow, 2017). It should be noted that while there is a concept similar to OCB, its nature is distinct: Compulsory Citizenship Behaviors (CCBs). CCBs refer to involuntary extra-role behaviors that arise under external pressure, not from the individual’s genuine goodwill. According to existing literature, various positive organizational and managerial factors can positively influence OCB. However, these factors may inadvertently pressurize employees, compelling them to display what appears to be OCB, but is in fact imposed. Such behaviors are termed as CCBs (Yildiz et al., 2023). In another study, Yildiz et al. (2022) examined the CCBs, anger, and moral disengagement levels of nurses during the COVID-19 pandemic. They found that when nurses are subjected to CCBs, they might harbor feelings of resentment toward the organization. This can drain employees’ positive energy and resources, and potentially compromise their moral decision-making mechanisms. In essence, imposing extra behaviors upon employees without their genuine willingness can be more detrimental than beneficial to organizations.

Another concept, akin to OCB and gaining traction in recent organizational behavior studies, is Constructive Deviant Workplace Behaviors (CDWB). While both are similar in that they exceed typical role expectations, OCB has a more passive nature, necessitating employees’ adherence to organizational and managerial norms and rules. In contrast, constructive deviance demands proactive actions from employees that may contravene norms. This suggests that employees exhibiting constructive deviance tend to be more risk-prone than their peers (Yildiz et al., 2015).

The above comparison helps clarify the characteristics of OCB. According to existing literature, OCB has been defined from a variety of perspectives (Suprapty Hidar et al., 2023). However, after reviewing these definitions, most scholars agree that OCB represents behaviors demonstrated by employees which, although not required for their current task or role, contribute to the organization’s operations and growth (Al-Ahmadi and Mahran, 2021). Examples of OCB in the workplace may include assisting coworkers and initiating improvement measures. Consequently, understanding why employees engage in OCB is both necessary and insightful. Educators have positive perceptions of organizational citizenship, with behaviors including suggesting improvements for the university, voluntarily assisting new lecturers, and dedicating their personal time to enhance the performance of their students and the university (Khalid et al., 2021; Bastian and Widodo, 2022).

On the other hand, CWB refers to actions that can be detrimental to an organization or its members. This type of behavior has garnered increasing attention from scholars and managers due to its potential negative impacts on businesses (Reizer et al., 2020). Some scholars adopts the psychological contract theory to explain the relationship between workplace ostracism and employees’ CWB in the tourism industry of China, found that understanding the effects for employees who are working in a cultural context that attributes high value on relationships and implicit psychological contracts (Li and Khattak, 2023). It is important to emphasize the defining characteristics of CWB: it is goal-oriented, as employees intentionally partake in harmful behavior (Akbari et al., 2022). As such, the repercussions of this behavior can significantly affect a wide range of stakeholders, including employees, coworkers, customers, and others.

## Reasons for research OCB and CWB

Why are scholars so interested in studying OCB and CWB? There are two primary reasons. First, both OCB and CWB fall under a broad definition of work performance that extends beyond assigned tasks (Neale, 2019). When assessing an employee’s performance, managers take these behaviors into account. Second, both OCB and CWB influence individual and organizational effectiveness and productivity (Susnienė et al., 2021). OCB is typically associated with positive outcomes such as improving coworker/managerial activities, efficient utilization of resources, employee retainment, while CWB is generally linked to negative outcomes like theft; destruction of property; sabotage; misuse of information, time and resources (Shah et al., 2022). At present, much interest has recently been paid to employee extra-role work behaviors (i.e., OCB, CWB) that are outside the technical core (i.e., task performance) but “shape the organizational, social, and psychological context that catalyzes task activities and processes” (Macias et al., 2023).

Some researchers have sought to more comprehensively explain the origins of OCB and its impact on organizational development. Some hypothesize that OCB leads to improved organizational performance and outcomes (Romi et al., 2019). Numerous studies have tied perceptions of unfair treatment to CWB actions, such as Siswanti et al.'s (2020) study, which employed organizational fairness theory and leader-member exchange theory to elucidate the connection. Just like Fein et al. (2023) study, who found that both OCB and CWB can be consequent behaviors following perceptions of distributive organizational injustice perceived as inequity.

According to Liu et al. (2023), employees’ turnover intention is positively related to their subsequent CWB, and permanent workers are less likely to engage in CWB compared to temporary workers because of the former’s higher organizational affective commitment. As Talaeipashiri (2016) stated, aggression may occur within the organization and could be targeted at certain individuals or the organization as a whole. Thus, we can conclude that organizational CWBs refer to actions directed at the organization itself, such as theft or use of violence, whereas interpersonal CWBs refer to actions directed at individuals within the organization, such as rudeness toward coworkers.

## Impact on the organization

Due to the importance of employee performance, OCB is crucial to an organization. Previous research has shown that organizations benefit from employee contributions that go above and beyond the formal job requirements, also known as OCB (Organ, 2018). Scholars strive to explain the positive effects of OCB from a broader research perspective (Vagner et al., 2022). For instance, OCB presents commitments that reasonable in nature and when totaled after some time and people, may upgrade the execution by greasing up the building the mental texture of the association, decreasing erosion, and/or expanding productivity (Guntuku et al., 2020). Furthermore, some scholar’s studies have highlighted the relationship between OCB and employee, they found that OCB has a significant and negative impact on intention to leave. When an employee has performed better OCB, it will lead to a lower intention to leave the organization (Abror et al., 2020).

The majority of CWBs involve proactive actions that intentionally or voluntarily harm an organization and its stakeholders, such as clients, colleagues, and supervisors (Liu et al., 2023). CWBs specifically include intentionally failing to perform work duties properly, engaging in workplace deviance, or engaging in behaviors that violate organizational policies and procedures (Mert, 2023). The most critical aspect of CWB is that they must be intentional and purposeful, not accidental (Kraak et al., 2023). Thus, when a worker chooses and engages in such harmful behavior, they do so with a conscious intent.

Actually, CWB are generally assimilated to “arbitrary behaviors performed by employees that overshadow the accepted norms of the organization and might then inflict pernicious shocks on the body of the organization and lead to extensive economic and psychological losses” (Akbari et al., 2022). It can be seen as a mechanism for employees to engage in deliberate behavior to restore perceived fairness in their transactions with the organization (“I am not paid enough, so I will work less”). According to researcher’s study, CWB is prevalent in the workplace and is regarded as one of the most pressing challenges encountering current organizations, costing them billions annually (Macias et al., 2023).

## Behavioral manifestations of OCB

OCBs are defined as “individual behavior that is discretionary, not directly or explicitly recognized by the formal reward system and promotes the effective functioning of the organization as a whole” (Organ, 1988; Fein et al., 2023). A multitude of strategic Human Resource Management issues—such as talent management, employee engagement, organizational climate, organizational effectiveness, turnover intentions, and organizational commitment—are intricately connected with human behavior-related psychological issues (Ren et al., 2023). Among all of these antecedents HRM practices play the most vital and challenging role in enhancing employees OCB (Sultana and Johari, 2023). As a result, organizations are keen to maintain industrial harmony through the identification of sociable behavioral skills, underscoring the practical relevance of this research.

Simultaneously, the growing interest in the study of OCB indicates that even positive behaviors can lead to negative outcomes. Several studies suggest that organizational citizenship behavior can be time-consuming (Reizer et al., 2020), potentially distracting workers from their core tasks and leading to employee burnout (Klotz et al., 2018). Specifically, some researchers have proposed that attachment acts as a personality regulator in the relationship between OCB and Work-Family Facilitation (WFF) (Reizer et al., 2020). Numerous studies show that attachment orientation can illuminate how individuals connect with others and foster healthy interpersonal relationships (Gazder and Stanton, 2023). These orientations, which consider fundamental personality tendencies, provide a theoretical foundation and a set of empirically validated data in the social and personality domains, and personality traits have a significant impact on direct and indirect organizational citizenship behaviors for the environment (Szostek, 2021).

In general, OCB is a crucial factor for organizational development (Somech and Ohayon, 2019), contributing to the creation of a psychosocial work environment that supports the organization’s core activities (Organ and Ryan, 1995). Regarding the direction and typology of OCB, several models have been developed since the construct’s inception (Turner and Connelly, 2021).

In Organ’s (1988) research, he identified five different types of behavior to exemplify organizational citizenship behavior: altruism, conscientiousness, sportsmanship, courtesy, and civic virtue (Atatsi et al., 2021).

### Altruism

Altruism entails discretionary assistance provided to peers or colleagues concerning job-related tasks, such as helping newcomers and freely dedicating time to others. While typically directed at individuals, it enhances group efficiency by improving individual performance (Dipaola and Hoy, 2005). In essence, altruism is “a motivational state with the ultimate goal of increasing another’s welfare” (Ma et al., 2018).

### Conscientiousness

Conscientiousness alludes to behavior that surpasses the minimal expected levels, like efficient time use and exceeding base expectations, thereby enhancing both personal and group efficiency (DiPaola and Hoy, 2005). Notably, conscientiousness is among the Big Five personality traits, epitomizing diligence and self-discipline. It has been identified as a consistent predictor of academic achievement (Icekson et al., 2020). Additionally, Abbas and Raja (2019) found conscientiousness to be the most influential predictor of problem-solving coping in response to stressors.

### Sportsmanship

Sportsmanship is an individual’s capacity to endure suboptimal situations without complaints (Lan, 2018), such as refraining from unnecessary grievances, thereby enhancing productive organizational time (Dipaola and Hoy, 2005). Despite its importance, sportsmanship has garnered limited attention in academic literature. Organ’s definition appears narrower than the broader implications of the term. For instance, “good sports” not only tolerate inconveniences but also maintain positivity despite setbacks, do not take offense easily, sacrifice personal interests for collective good, and handle rejection gracefully (Podsakoff et al., 2000). Puspitasari et al. (2023) suggest that sportsmanship enables teachers to tolerate imperfect organizational conditions without dissent. High sportsmanship fosters a positive climate, promoting collaboration and creating a harmonious work environment.

### Courtesy

Courtesy is characterized as polite and thoughtful actions toward colleagues. Employees exhibiting courtesy consciously evade causing issues for others, thereby reducing managerial burdens and amplifying organizational performance (Faajir et al., 2021). Such behavior is proactive, preventing issues rather than addressing existing problems (Magdalena, 2014). Examples include giving advance notices and reminders, which helps avert issues and ensures productive time utilization (Dipaola and Hoy, 2005). In essence, courtesy fosters positive relations among peers, crafting a conducive and amiable work setting (Oamen, 2023).

### Civic virtue

Civic virtue encompasses behaviors emphasizing participation in overarching organizational issues, like committee work and voluntary attendance at events, bolstering the organization’s interests (Dipaola and Hoy, 2005). Robbins and Judge (2015) equate civic virtue with responsible behavior, which includes following organizational changes, suggesting improvements, and safeguarding organizational resources. Civic virtue implies that organizations empower employees to enhance their work quality (Puspitasari et al., 2023). Broadly, it signifies an employee’s inclination to represent and elevate their organization’s image positively (Oamen, 2023).

Contemporary literature explores other distinctions within OCB, although many of these dimensions are still applicable. In the early 1990s, researchers began differentiating between Organizational Citizenship Behavior—Individual (OCBI) and Organizational Citizenship Behavior—Organizational (OCBO) (Smith et al., 1983). OCBIs involve helping behaviors directed toward other individuals (e.g., assisting a sick coworker), while OCBOs encompass actions directed at the entire organization, such as participating in a voluntary company fundraiser. Proponents of this perspective argue that OCBI and OCBO are distinct variables with unique antecedents and motivators and that they are associated with job satisfaction in different ways (El-Kassar et al., 2021; Rahman and Karim, 2022).

## Behavioral manifestations of CWB

The means and likelihood of employee retaliation-based behaviors as reactions to poor leadership and management have been noted extensively as behavioral manifestations of Counterproductive Work Behaviors (CWB) (Fein et al., 2023). Individual CWBs refer to actions directed against individuals within the organization, while organizational CWBs refer to actions against the organization as a whole. The study of deviant workplace behavior by Robinson and Bennett (1995) provides evidence for this interpretation.

Several researchers have examined the connections between CWB and occupational stressors. Some researcher found that perceived increases in workload were positively related to increased exhaustion after work, psychosomatic symptoms, and to spillover effects at home, even after controlling for negative affect (Rodríguez, 2019). The same as Lenz et al. (2023) study, whose research suggests that when exposed to stressors, individuals take longer breaks, or work slower than necessary (i.e., show CWB) as a strategy to avoid further resource loss. The work stress/mood/CWB model developed by Fox et al. (2001) suggests that CWB is an instinctive emotional response to workplace stressors. According to Spector and Jex (1998), workplace stressors are understood to pose threats to health and to lead to negative emotional responses such as anger and anxiety. Furthermore, some scholars argue that job insecurity is associated with CWB behavior. Many organizations face restructuring and downsizing, especially in today’s uncertain and volatile economic climate, which can heighten employee anxiety and stress (Pu et al., 2023).

Here is a comprehensive explanation of the five components of CWB. Mistreatment of others is considered individual counterproductive behavior (CWB), whereas deviant behavior, destructive behavior, withdrawal behavior, and theft are classified as organizational counterproductive behaviors (CWB).

### Abuse against others

Abuse against others within an organization involves an individual’s behavior that is harmful to their coworkers (Bal, 2021). These behaviors can inflict physical harm, such as humiliation, contempt, insulting remarks, or intimidation, or psychological harm, such as neglect and hindering effective work. Simultaneously, it should be stressed that since direct and overt physical violence is rare within organizations, many researchers focus on non-violent behaviors. The concept of abuse in this context is closely related to notions of incivility, emotional abuse, workplace bullying, and psychological siege, as outlined in the relevant literature. In other words, within the context and scope of CWB research, the study focuses on individuals who engage in these actions (To and Huang, 2022).

### Production deviance

The component of production deviance includes behaviors such as not deliberately and properly performing the tasks in the job description of the employee, making mistakes, performing poorly, slowing down and obeying the instructions (Bal, 2021). A summation of items reflecting “interpersonal and organizational deviance” should indicate the participation levels of each form of deviance (Fleming et al., 2022). Early work in CWB focused on what was characterized as employee deviance, falling into categories of product deviance, property deviance, political divisions, and personal aggression; while deviance has been characterized as “violating behaviors,” which are those that benefit self, those that benefit the organization in an unethical manner, or destruction to exact revenge (Allen, 2023).

### Sabotage

Sabotage involves the intentional and deliberate destruction (such as arson or property damage) or damage of organizational assets (like equipment) by employees in an effort to reduce productivity (Spector et al., 2006; Kim and Jo, 2022). This vandalism can be traced back to the machine destruction during the workers’ movement following the Industrial Revolution, and can be seen as an extension or derivation of that act. In some studies, destructive behavior is interpreted from a broader perspective and is considered as negative behaviors based on employees’ personal interests, such as damaging organizational functions, disrupting or altering organizational order, creating and spreading negative rumors within the organization, slowing production, or harming customers and employees (Skarlicki et al., 2008; Szostek, 2022). Several factors contributing to the emergence of destructive behavior include anger or hostility, responses to unfairness, the desire for personal gain, resistance to organizational change, and the need for approval from coworkers (Wiseman and Stillwell, 2022).

### Withdrawal

Withdrawal includes reduce the working time below the minimum necessary to achieve the goals (for example, extending breaks, unjustified dismissals). Different from other forms of CWB, the employees engaged in withdrawal were characterized by a lower level of emotional exhaustion (Szostek et al., 2020). Withdrawal is behavior where an employee attempts to avoid a situation rather than harming the organization and its members thus, this type of behavior is used as a passive way to influence the organization by withholding effort usually used to produce for the organization. At the same time, looking at the description of production deviance there is a noticeable similarity between the categories, but as previously stated, withdrawal is more passive in that it involves withdrawing effort systematically (Van der Westhuizen, 2019).

### Theft

Employees commit theft with the intention to harm organizations or individuals (Sackett et al., 2006). It is a form of instrumental aggression (mainly toward the organization) motivated by the will to: obtain approval, help colleagues, equalize conditions and protect oneself in case of harmful actions of superiors (Szostek, 2022). Many employees may view theft from the organization as non-aggressive due to financial needs, dissatisfaction with the job, or a sense of being treated unfairly (Bal, 2021). In these instances, employees do not intend to use or sell the stolen items but aim to harm the organization’s economic interests.

## The influencing factors of OCB and CWB

An individual’s inherent and immutable personality has a more stable and lasting impact on OCB/CWB (Aspan et al., 2019). Previous research has elaborated on why intrinsic motivation theory can influence employees’ propensity to engage in civic behavior. Intrinsic motivation refers to the internal factor of employee self-satisfaction (Runge et al., 2020; Schattke and Marion-Jetten, 2022). Since OCBs are less likely to be formally rewarded than prescribed work behaviors, they are most likely to be driven by internal incentive channels (Dermawan and Handayani, 2019; Ren et al., 2022).

Personality traits can influence how individuals perceive and respond to diverse motivations (Clark, 2010; Reizer et al., 2020). According to Neale’s (2019) study, the findings suggest that that the intentionality behind job crafting behaviors is predicted differentially by individual needs as well as personality traits (the dark triad and conscientiousness). Bright job crafting is more associated with engagement in OCBs while dark job crafting is more associated with engagement in CWBs. Related research demonstrates that organizational commitment is the most influential factor affecting OCB. High organizational commitment is related to high OCB and employee performance, low absence rates, and fewer delays (Nurjanah et al., 2020).

Furthermore, it is believed that organizational commitment is positively related to perceived organizational support. When employees feel respected and supported for their roles, organizational commitment increases (Lambert et al., 2017). This bond can be strengthened in numerous ways. Leadership has a significant effect on the perception of organizational support (Wang et al., 2021). Specifically, Delegach et al. (2017) found that transformational leadership is positively associated with organizational commitment, whereas transactional leadership is positively associated with commitments to safety and the organization’s mission. Given the strong emphasis on transformational leadership practices in encouraging OCBs, these findings are intriguing. It’s possible that organizational commitment may increase if transactional leaders are better equipped to instill organizational values in employees.

Some scholars believe that job autonomy may have positive effects on organizational performance. Job autonomy is defined as the extent to which the job offers employees the freedom to make choices about what, when, and how they perform their work. Greater job autonomy reduces limitations from other job factors and improves individuals’ job performance (Matteson et al., 2021). These contradictory findings and a contingency perspective suggest that the relationships between job autonomy, OCB, and organizational performance may depend on organizational circumstances (Park, 2018).

## Conclusion

From the comprehensive literature review, we observe various research perspectives and conclusions on deviant behaviors. In studying constructive and destructive deviant workplace behaviors, scholars have refined a general classification of workplace deviance. Using precise definitions of terms, they have analyzed antecedent factors, constructed various models or frameworks, and proposed feasible measures. This literature review aids in further summarizing the relevant content concerning OCB and CWB.

In this paper, previous scholars’ conclusions shed light on the propositions. In general, this paper provides a succinct overview of previous research on deviant behaviors, with a particular focus on OCB and CWB as well as their various aspects. It discusses personality, organizational commitment and job autonomy, three concepts intrinsically related to OCB/CWB, and how they function. This section underscores the impact that CWB and OCB have on organizational performance. Each aspect of CWB and OCB is also detailed within this study for relevance. The literature review offered above allows us to envision an optimal portrayal of organizational performance, and this theoretical framework can be beneficial in terms of practitioners and researchers. Within organizations, employees should exert additional effort and be open to adopting new work methods, while leaders should provide comprehensive support, effectively implement employees’ suggestions, set high standards, and commit more resources and energy to work-related matters rather than traditional management and rigid control. Given sufficient trust, employees are more likely to engage in cooperative behaviors, such as assisting coworkers and performing actions that benefit the group. Consequently, the costs associated with hiring, selecting, and integrating new coworkers should be reduced. Although this is not an empirical paper, the compilation of previous research findings constitutes a significant contribution to guiding managerial actions in organizations. This paper can serve as a guide for organizations seeking to improve their employees’ organizational performance and curtail the occurrence of negative behaviors.

The limitations of this paper are manifold. While the primary focus was on OCB and CWB, the intricate relationships among OCB, CWB, and deviant workplace behaviors were not fully explored. Moreover, the study centered on just three determinants: personality, organizational commitment, and job autonomy, assessing their influence on OCB/CWB. Future studies might consider a broader range of individual, task, and organizational antecedents and delve into potential indirect effects, such as moderator impacts, on OCB and CWB. Furthermore, this research did not narrow down to specific industries or professions, suggesting that subsequent research, when tailored to distinct sectors or job roles, might yield recommendations with heightened relevance and applicability.

## Author contributions

All authors listed have made a substantial, direct, and intellectual contribution to the work and approved it for publication.

## Conflict of interest

The authors declare that the research was conducted in the absence of any commercial or financial relationships that could be construed as a potential conflict of interest.

## Publisher’s note

All claims expressed in this article are solely those of the authors and do not necessarily represent those of their affiliated organizations, or those of the publisher, the editors and the reviewers. Any product that may be evaluated in this article, or claim that may be made by its manufacturer, is not guaranteed or endorsed by the publisher.
